# The potential application of probiotics and prebiotics for the prevention and treatment of COVID-19

**DOI:** 10.1038/s41538-020-00078-9

**Published:** 2020-10-05

**Authors:** Amin N. Olaimat, Iman Aolymat, Murad Al-Holy, Mutamed Ayyash, Mahmoud Abu Ghoush, Anas A. Al-Nabulsi, Tareq Osaili, Vasso Apostolopoulos, Shao-Quan Liu, Nagendra P. Shah

**Affiliations:** 1 Department of Clinical Nutrition and Dietetics, Faculty of Applied Medical Sciences, The Hashemite University, P.O. Box 330127, Zarqa, 13133 Jordan; 2 Department of Basic Medical Sciences, Faculty of Medicine, The Hashemite University, P.O. Box 330127, Zarqa, 13133 Jordan; 3 Department of Food Nutrition and Health, College of Food and Agriculture, United Arab Emirates University, Al Ain, UAE; 4 Department of Nutrition and Food Technology, Jordan University of Science and Technology, P.O. Box 3030 Irbid, Jordan; 5 Department Clinical Nutrition and Dietetics, University of Sharjah, Sharjah, UAE; 6 Institute for Health and Sport, Victoria University, Melbourne, VIC Australia; 7 Department of Food Science and Technology, Faculty of Science, National University of Singapore, S14 Level 5, Science Drive 2, Singapore, 117542 Singapore; 8 Food and Nutritional Science, School of Biological Sciences, The University of Hong Kong, Pokfulam Road, Hong Kong, Hong Kong

**Keywords:** Applied microbiology, SARS-CoV-2

## Abstract

COVID-19 is a pandemic disease caused by the novel coronavirus severe acute respiratory syndrome coronavirus 2 (SARS-CoV-2). This new viral infection was first identified in China in December 2019, and it has subsequently spread globally. The lack of a vaccine or curative treatment for COVID-19 necessitates a focus on other strategies to prevent and treat the infection. Probiotics consist of single or mixed cultures of live microorganisms that can beneficially affect the host by maintaining the intestinal or lung microbiota that play a major role in human health. At present, good scientific evidence exists to support the ability of probiotics to boost human immunity, thereby preventing colonization by pathogens and reducing the incidence and severity of infections. Herein, we present clinical studies of the use of probiotic supplementation to prevent or treat respiratory tract infections. These data lead to promising benefits of probiotics in reducing the risk of COVID-19. Further studies should be conducted to assess the ability of probiotics to combat COVID-19.

## Introduction

The evolution and emergence of viruses have significantly increased in the last two decades because of their rapid mutation. The emergence or re-emergence of viruses is attributable to several factors including increased numbers of immunocompromised patients, climate change, the absence of anti-viral agents, the increased geographical movement of people and goods, and genetic modification of viruses^1,2^. Respiratory infections represent a major cause of death and disability worldwide in both developing and developed countries^3^. It has been estimated that acute respiratory infections including pneumonia, influenza, enterovirus, adenovirus, and respiratory syncytial virus infections are responsible for millions of deaths every year. In addition, they have a substantial economic and social impact because of their associated high hospitalization rate, high medical costs, and losses of productivity associated with time missed from work or school. It has been estimated that the annual cost of viral respiratory tract illnesses is approximately US $40 billion in the United States^1^. The majority of these infections are caused by more than 200 different types of viruses that may contain RNA or DNA as genetic material. Infections related to RNA viruses are more remarkable than those caused by DNA viruses. In particular, coronaviruses represent a highly important emerging RNA virus family^2^.

## Coronavirus disease 2019 (COVID-19)

Severe acute respiratory syndrome coronavirus 2 (SARS-CoV-2) is a novel coronavirus that causes coronavirus disease (COVID-19) in humans, a respiratory infection that was first reported in Wuhan, China in December 2019. This SARS-related coronavirus is a member of the zoonotic beta-coronavirus family^4,5^. SARS-CoV-2 is an enveloped virus with a single-stranded positive sense RNA genome^6^. Coronaviruses are named for their crown-like shapes associated with their long surface spikes^7^. Coronaviruses are hosted by humans and several other vertebrate reservoirs such as camels, bats, masked palm civets, mice, dogs, and cats^8,9^. It has been suggested that COVID-19 was initially hosted by bats and then transmitted to humans via wild animals; however, the subsequent spread of the virus occurred through human-to-human transmission^7^.

Coronaviruses may cause respiratory, gastrointestinal (GI), and neurologic disorders^8^. Most of the identified coronaviruses cause mild human disease excluding SARS-CoV-1 and Middle East respiratory syndrome coronavirus (MERS-CoV), which are highly pathogenic viruses associated with severe infections and fatalities^9,10^. SARS-CoV-1 appeared in 2002 in China, whereas MERS-CoV was identified in 2012 in Saudi Arabia^4,8^. Although SARS-CoV-2 is more transmissible than MERS-CoV and SARS-CoV-1, it has lower fatality rates than either virus^8^. COVID-19 is highly pathogenic, and the number of affected patients has drastically increased globally. Therefore, COVID-19 was declared pandemic by WHO, which confirmed at least 32.5 million cases and more than 986.000 deaths through September 26, 2020^10^.

The incubation period for COVID-19 is 1–14 days^10^. The clinical manifestations of COVID-19 are variable, ranging from asymptomatic to severe illness. Asymptomatic patients can serve as sources of disease dissemination^5,8^. Common symptoms of COVID-19 include fever, dry cough, shortness of breath, myalgia, and fatigue. Headache, rhinorrhea, sneezing, sore throat, loss of odor and pneumonia are other reported symptoms of COVID-19^8^. Other uncommon manifestations of the disease include gastrointestinal symptoms such as diarrhea, nausea, vomiting, and abdominal pain^7,8^.

The majority of cases are self-limiting and result in complete recovery^5^. Conversely, SARS-CoV-2 can cause severe infections and result in septic shock, acute respiratory distress syndrome, acute cardiac injury, acute kidney injury, and multi-organ failure, which necessitate intensive care unit admission. Extremely severe cases of COVID-19 can lead to death^5,7,8^. Adults and children usually develop mild self-limiting disease. Meanwhile, more severe COVID-19 can occur in elderly people with underlying medical conditions such as cardiovascular diseases and diabetes^5^. Pregnant women usually develop a disease status similar to that in non-pregnant adult patients. Although COVID-19 was initially considered uncommon in children^11^, increasing numbers of pediatric cases have been reported worldwide, suggesting that children have the same susceptibility to COVID-19 as adults but exhibit mild or asymptomatic disease^12^.

A history of contact with infected patients within 2 weeks should raise the suspicion of COVID-19 infection^7^. Real-time reverse-transcription–polymerase-chain-reaction is the gold standard diagnostic tool for COVID-19. Moreover, chest computed tomography is another modality supporting the clinical diagnosis of COVID-19^7^.

COVID-19 is mainly transmitted from person-to-person via sneeze- or cough-induced respiratory droplets from the mouths or noses of infected persons^5,8^. Disease transmission through the eyes has also been suggested. Contact with surfaces contaminated by the virus is another mode of COVID-19 transmission^4,9^. Recently, SARS-CoV-2 was detected in stools, suggesting the possibility of fecal–oral transmission^13^. This was later confirmed in some cases in the US and China which indicated that SARS-CoV-2 can multiply in both respiratory and digestive tracts^14^. Furthermore, the fecal samples of some infected patients were found positive for the RNA of SARS-CoV-2 after their respiratory samples became negative for the viral RNA^15^. It seems that COVID-19 infection negatively affect the anatomy and physiology of the GI tract for a long period and thus, attacking the gut microbiota^16,17^. Nowadays, a solid body of available evidence confirmed that the gut microbial community of COVID-19 patients had been changed. It was obvious that growth of opportunistic pathogens and reduction of beneficial bacteria in gut microbiota positively correlated with the severity of COVID-19 infections^18,19^.

## Gut microbiota

Vaccines are promising treatments for preventing viral infectious disease; however, their efficacy can be limited by mutations in RNA viruses, as observed for the influenza virus as a representative pathogenic virus^20,21^. This increases the risk of infection, making these viruses serious threats to public health because of recurrent widespread outbreaks.

The microbial communities (bacteria, fungi, archaea, viruses, and protozoa) in the human GI tract, lungs, skin, and mouth exist in a commensal relationship with host cells, thereby playing a major role in human health^22,23^. The commensal bacteria (1 × 10^13^ CFU) that are present in the GI tract are equivalent to the number of human cells^24^. This colonization starts shortly after birth and their profiles and numbers stabilize by the age of 1 year with more than 1000 bacterial species^25,26^. The GI microbiota has the ability to interact with human cells, including specific immune cells. These interactions produce different health benefits in the host including regulating GI motility; activating and destroying toxins, genotoxins, and mutagens; transforming bile acid and steroids; producing vitamins; absorbing minerals; metabolizing xenobiotic substances; influencing intestinal permeability and barrier functions; and modulating mucosal and systemic immunity; as well as beneficial effects on the skin and upper respiratory tract^26,27^.

Recently, the presence of beneficial microbes was reported in the upper (nasal cavity, nasopharynx, oropharynx, and larynx above the vocal cords) and lower respiratory tracts (larynx below the vocal cords, trachea, bronchi, and bronchioles and alveoli of the lungs) of both healthy people and those with pulmonary diseases such as cystic fibrosis and chronic obstructive pulmonary disease^20,28^. The microbiota populated the lungs mostly via the upper respiratory tract or diffusion along the mucosal surface^28,29^.

These beneficial microorganisms compete with pathogens concerning the colonization of human cells in different organs to promote host health. This requires high numbers of beneficial microorganisms, and any imbalance or disruption of this system may cause dysbiosis, which can allow pathogens to cause diseases such as respiratory tract infections^20,22^. Dysbiosis can also be caused by long-term antibiotic use. Therefore, probiotics are also usually recommended for patients who have recently used antibiotics for treating any disease. Other causes of dysbiosis in the human GI tract include exposure to toxins, stress, disease, insufficient diet, and age^26^.

## Gut-lung axis and COVID-19

The gastrointestinal tract and lung are among the body compartments that host microbiota; however, the lung has a small number of microbiota when compared to that of the gut^30^. There is accumulating evidence that bidirectional communications exist between gut and lung, which is called the gut-lung axis. This bidirectional crosstalk is involved in the support of immune homeostasis^31^. It is believed that the gastrointestinal inflammation results in lung inflammation through this connection^32^. The exact mechanism underlying this inflammatory shift from the gut to the lung is not yet completely revealed; however, dysbiosis of gut and lung microbiota is one of the implicated factors in this event. It has been shown previously that dysbiosis of gut microbiota is linked with several respiratory pathological conditions^32,33^, and shifts in the composition of the lung microbiota toward the gut microbiota have been observed in several respiratory disorders^30,34^. One of the suggested mechanisms behind the bidirectional interaction between lung and gut microbiota systems is that increased permeability of the GI tract allows the leakage and migration of the gut microbiota to the lung, modulating its microbiota and thus its immune responses^30^. Furthermore, gut microbial components and metabolites like lipopolysaccharides (LPS) and short-chain fatty acids (SCFA), respectively, are also involved in this gut-lung bidirectional communication. Additionally, blood- or lymphatic-mediated circulation of immune cells or inflammatory mediators from the GI tract to the lung results can in lung inflammatory responses^30,35^.

In addition to the most frequently described respiratory symptoms such as fever, cough and severe respiratory syndrome caused by COVID-19 infection, it has also been reported that patients exhibited GI symptoms including diarrhea, vomiting, nausea, loss of appetite, GI bleeding, and abdominal pain^36^. It was found that COVID-19 patients with GI symptoms such as diarrhea experienced more severe respiratory disorders than those without GI symptoms^37^. Although the impact of the gut on lung health is well established, the available knowledge about the opposite role of the lung on the gut health is still scarce. Therefore, it is unknown why would COVID-19 influence the GI tract integrity. Dysbiosis is potentially one of the contributing mechanisms. Little knowledge is available about the effect of lung microbiome on the gut one. Acute lung injury mediated-lung dysbiosis was associated with blood-mediated modulation of the gut microbiota^38,39^, and the gut microbiota population is modulated in cases of pulmonary allergy^40^. As a result, COVID-19 may induce lung microbiota disruption that modulates the GI tract microbiota, resulting in GI tract symptoms.

Furthermore, studies revealed that the GI symptoms generated in patients infected with COVID1-9 might be attributed to the damaged tissues and organs caused by the immune responses^41^. Alternatively, angiotensin-converting enzyme 2 (ACE2) is the main host cell receptor of COVID-19^42,43^. ACE-I and ACE-II are crucial enzymes that play a significant role in regulating blood pressure via the biochemical renin-angiotensin-aldosterone system (RAAS) pathway^44^. Besides the lung, ACE2 is also expressed by the intestines, and direct colonization of the gut ACE2 receptors through the ingestion of the virus is potentially responsible for the gastrointestinal tract symptoms associated with COVID-19. Instead of that, dysfunction of apoptosis pathways in the intestine due to respiratory infections^45^ is another proposed explanation for COVID-19-associated GI tract symptoms. Additionally, it is still likely COVID-19-related GI tract symptoms might result due to the fact that GI tract and respiratory tracts share the same embryonic origin, and thus they are structurally alike and interact similarly in physiological and pathological conditions^46^. All these suggested mechanisms can work individually or collectively to induce GI tract disturbances associated with COVID-19.

To date, no vaccine has been developed for COVID-19, nor is any curative therapy available. Most available treatments aim to alleviate symptoms, and mechanical ventilation is used in cases of severe disease. Some anti-viral, anti-inflammatory and anti-malarial drugs have been applied to treat COVID-19; however, none of these medications have been approved as effective curative treatments against COVID-19^8^. Therefore, other safe strategies such as probiotics and prebiotics could be applied to prevent or treat COVID-19.

## Probiotics and prebiotics

Probiotics are live microorganisms that confer a beneficial physiological effect on the host when administered at adequate amounts. Some lactic acid bacteria that can be found in different fermented foods such as yogurt, cheese, and pickles are generally recognized as safe and classified as probiotics because of their health benefits^47^. It was suggested that probiotics should be consumed daily at doses of 10^8^ to 10^10^ CFU to produce health benefits in humans. The approved health benefits include reducing symptoms of lactose intolerance by improving lactose digestion, inhibiting the initiation of allergic diseases, maintaining intestinal pH, preventing or treating ischemic heart syndromes, reducing blood cholesterol levels, producing vitamins B, improving the bioavailability of dietary calcium, and boosting immune activity. Meanwhile, other potential health benefits such as the treatment of acute diarrheal diseases and prevention of cancer and tooth decay require additional research for validation^22^.

Probiotics include bacteria such as *Lactobacillus acidophilus, L. amylovorus, L. brevis, L. bulgaricus, L. casei, L. cellobiosus, L. crispatus, L. curvatus, L. delbrueckii spp. bulgaris, L. fermentum, L. gallinarum, L. helveticus, L. johnsonii, L. lactis, L. paracasei, L. plantarum, L. reuteri, L. rhamnosus; Streptococcus thermophilus, Lactococcus lactis, Leuconostoc mesenteroides, Pediococcus pentosaceus, P. acidilactici, Bifidobacterium adolescentis, B. animalis, B. bifidum, B. breve, B. essensis, B. infantis, B. laterosporum, B. thermophilum, B. longum, Propionibacterium acidipropionici, P. freudenreichii, P. jensenii, P. thoenii, Enterococcus fecalis, E. faecium, Bacillus alcolophilus, B. cereus, B. clausii, B. coagulans, B. subtilis, Escherichia coli, Sporolactobacillus inulinus*; as well as yeast such as *Saccharomyces boulardii* and *S. cerevisiae*^48–50^. Probiotics have been proposed as antimicrobial agents against a large number of pathogenic and spoilage bacteria. However direct and indirect anti-viral activity was recently reported for some probiotic strains^51^.

Prebiotics were initially defined as “non-digestible food ingredients that beneficially affects the host by selectively stimulating the growth and/or activity of one or a limited number of bacteria already residing in the colon”^52^. The prebiotics definition has been modified several times and finally it was proposed as ‘substrates that are selectively utilized by host microorganisms conferring a health benefit’^53^. Similar to probiotics, prebiotics can be delivered into microbially colonized body sites by oral administration to reach the intestines, or by a direct way to the vaginal tract and skin^53^. Prebiotics include fructans, oligosaccharides, arabinooligosaccharides, isomaltooligosaccharides, xylooligosaccharides, resistant starch, lactosucrose, lactobionic acid, galactomannan, psyllium, polyphenols and polyunsaturated fatty acids^53–55^. The health benefits of prebiotics to the GI tract including inhibition of pathogens and stimulation of immune system are due to their ability to modulate the composition and activity of human microbiota^55^. However, to date, there is no information directly linking prebiotics to COVID-19 infections in any way, although a indirect effect may be hypothesized.

## Probiotics and immune modulation

Probiotic bacteria have been shown to have a number of beneficial immune and health effects. They not only enhance the bioavailability of nutrients and moderate health, they are also involved in regulating the bacterial ecosystem and module immune cells^56^.

Dendritic cells (DC) play a key role in immune homeostasis in the healthy intestine. DCs are key antigen presenting cells which take up antigens (i.e. viral, cancer) and present small antigenic peptides on their surface to prime T cells towards pro-inflammatory (Th1) or anti-inflammatory (Th2) phenotypes. DC in an immature state can lead to deletion of T cells or stimulation of regulatory T cells^57^. The gut microbiota are able to drive DC to prime these cells. In fact, *L. reuteri* and *L. casei*, stimulate IFN-gamma production, and activate pro-inflammatory Th1 cells^58^. Likewise, oral administration of *B. infantis* in mice stimulate DCs which suppress Th2-biased responses^59^ and stimulate Th1 pro-inflammatory responses which is required for virus elimination.

Monocytes are present in the peripheral blood which are amongst the first cells to be in contact with bacteria and viruses. They differentiate to tissue macrophages, where intestinal microbiota or ingested probiotics interact with them to secrete a number of cytokines. The pro-inflammatory cytokine, IL-12 is secreted by macrophages which stimulate natural killer cells and CD4^+^ Th1 cells to secrete IFN-gamma which are required for elimination of viruses^60^. In addition, probiotics *L. gasseri, L. delbrueckii* ssp. *bulgaricus*, *B. bifidum, L. acidophilus* strains induce IFN-alpha production by monocytes^60^. The probiotic *L. paracasei* DG increases TNF-alpha, IL-6, IL-8 of human monocyte cell line, THP-1^61^. Similarly, it was recently noted that *S. thermophilus* induced TNF-alpha, IL-6, IL-8 profile which is required for anti-viral effects^62–64^.

NK cells are important in the early immune response against viral infections, in particular through clearance of virus-infected cells. *Lactobacillus* probiotic strains are able to stimulate DCs to secrete IL-12, which in turn activates NK cells to secrete IFN-gamma, an essential cytokine for lung bacterial (*S. aureus*) and viral elimination^58,65^.

Probiotics such as *L. casei* can also interact with Toll-like receptors (TLR) on the epithelial cells, thereby, enhancing the production of cytokines that play a major role in improving the epithelial cells productivity and preventing their apoptosis which enhances their survival and proliferation during restoration^66,67^. Understanding the immune cell activation, cytokine profiles and immune modulation is crucial providing a clear path for managing viral infections.

## Probiotics and respiratory tract infections

Vaccines, antibiotics, and anti-viral medications have been regularly used for the prevention and treatment of bacterial or viral infectious diseases; however, the control of most infections has not yet been fully achieved. Antimicrobial resistance is progressively emerging among many pathogenic bacteria, viruses, parasites, and fungi^21,68^. Antibiotics are not recommended for treating viral infections because of their inactivity against viruses and disruption of the normal human microbiota. Therefore, other approaches have been developed for the treatment and prevention of bacterial or viral respiratory tract infections. These modalities include bacteriophages, antimicrobial peptides, and probiotics^51^. Probiotics exhibit potent antimicrobial activity against several pathogens. In the past two decades, probiotics have been proposed as antimicrobial agents against viruses causing respiratory tract infections^51^. There are different possible mechanisms of action supporting the activities of probiotics against respiratory viruses; however, the most probable mechanisms are modulation of the innate immune system and enhancement of acquired immune responses^1^. Based on previous studies of different viral infections, it is evident that the prevention of infectious diseases can be achieved by boosting and stimulating human immune activity through the consumption of healthy, balanced diets and the use of administrative supplements such as vitamins, minerals, fiber, and probiotics^51^.

Several studies have shown that probiotics are useful for preventing or treating respiratory tract infections caused by viruses such as influenza and syncytial viruses through enhancing the immunity of individuals via activating immunoglobulin A (IgA) secretion and boosting the activity of Peyer’s plaques, neutrophils, macrophages, natural killer cells, mesenteric lymph nodes, and intraepithelial lymphocytes^26,69^. Oral probiotic strains have been used to prevent or treat infections caused by influenza A, influenza H1N1, and respiratory syncytial viruses by minimizing the infectious symptoms, shortening the duration of infection, reducing the virus levels in the lungs or nasal washings, producing anti-viral components, promoting immune activity, and enhancing health by reducing body weight loss during infection^70–73^. Probiotic supplements containing *L. paracasei*, *L. casei*, and *L. fermentum* significantly reduced the incidence of influenza-like symptoms and upper respiratory infection in adults, who commonly experience colds ≥4 times per year. Treated adults exhibited significantly higher interferon (IFN)-γ levels in serum and higher soluble IgA levels in the GI tract than untreated adults or their own baseline results^74^.

In addition to oral probiotic administration, intranasal administration using nasal sprays and aerosolized formulations is considered an effective and non-invasive approach for distributing probiotics into cells in the lungs to modulate the microbiota and treat or prevent several viral infections^75–78^. Several probiotics species including *B. breve*, *L. pentosus*, *L. casei*, *L. plantarum*, *L. rhamnosus*, *L. delbrueckii* ssp. *bulgaricus*, *L. gasseri*, *L. reuteri*, *L. lactis*, and *L. brevis* have been intranasally or orally administrated^1,69^.

Harata et al.^79^ found that the intranasal administration of *L. rhamnosus* in mice infected with influenza H1N1 virus resulted in significantly diminished symptoms and higher survival rates than observed in control mice. They also reported that treated mice exhibited higher cytotoxic activity in the lungs, elevated mRNA expression of interleukin, tumor necrosis factor, and monocyte chemotactic protein. Marchisio et al.^80^ noted that the intranasal administration of *S. salivarius* effectively treated acute otitis media in children aged 1–5 years. Nasal administration of different strains of *L. rhamnosus* in mice conferred a protective effect against influenza and respiratory syncytial virus infection^81,82^. Kawase et al.^83^ reported that the oral administration of *L. gasseri* in mice infected with the influenza H1N1 virus significantly ameliorated clinical symptoms, reduced the viral load, and increased the mRNA expression of interleukins and IFNs. Youn et al.^84^ found that influenza virus-infected mice that were intranasally administered *Lactobacillus* species had higher survival rates than untreated mice.

## Probiotics, prebiotics, and COVID-19

The directly or indirectly positive impact of probiotics on the ACE enzymes is well stated^85^. During food fermentation, probiotics produce bioactive peptides with the capability to inhibit the ACE enzymes by blocking the active sites^86,87^. Moreover, the debris of the dead probiotic cells acted also as ACE inhibitors^88^. These findings suggest that probiotics could be a potential blocker to the ACE receptor that acts as a gateway for SARS-CoV-2 to attack GI cells. The concept of using drugs to block the ACE receptors as a treatment approach against COVID-19 was proposed by Fernández-Fernández^89^, despite the otherwise opinion expressed by Esler and Esler^90^. Imai et al.^91^ have stated a positive influence of using an ACE blocker to reduce respiratory distress syndrome.

Prebiotics may also have an excellent potential effect against COVID-19 by enhancing probiotics growth and survivability. Furthermore, prebiotics could have a direct effect on GI symptoms caused by COVID-19 via blocking the ACE enzymes. Yeh et al.^92^ systematically reviewed 12 studies that investigated the impact of prebiotic and probiotic supplementation on influenza infection. The authors concluded that the supplementation of probiotics and prebiotics could improve hemagglutination inhibition antibody titers following the influenza vaccination.

SARS-CoV-2 is a newly emerging virus that currently lacks curative treatments and vaccines. To date, no study has reported the use of prebiotics and probiotics to treat or prevent COVID-19, but the use of probiotics in the clinical treatment or prevention of COVID-19 could be a suitable strategy. So far, several registered trials that aim to investigate the efficiency of probiotics in treating COVID-19 patients are ongoing^93^. Some patients with COVID-19 exhibited intestinal microbial dysbiosis characterized by low numbers of different probiotic species such as *Bifidobacterium* and *Lactobacillus*. This is could be an indicator of their weak immunity, and therefore, it has been suggested that these patients require nutritional support and prebiotic or probiotic supplementation to re-normalize the intestinal flora balance and decrease the risk of infection^94^. COVID-19 is a novel disease, and humans have not acquired immunity against this disease. Meanwhile, the dietary pattern of patients is an essential factor for GI microbiota levels, diversity, structure, and function. Therefore, balanced diets including probiotics-containing foods and immunity-enhancing micronutrients such as polyphenols; vitamins A, C, and D; and minerals (mainly selenium and zinc) may alleviate the risk of COVID-19 infection^95^. Food sources of probiotics such as fermented products have a good potential to prevent COVID-19. In previous research, the consumption of fermented milk containing probiotic strains significantly reduced the incidence of upper respiratory tract infections among healthy infants, children, adults, and the elderly^96–99^.

Apparently, probiotic supplementation may be a suitable strategy given prior reports of the potential application of probiotics for preventing and treating several viral infections. These observations support the administration of probiotics to patients with COVID-19 despite the absence of solid evidence supporting that these treatments can prevent or treat this infectious disease. However, boosting the natural immunity of the population using probiotics before, during, or after COVID-19 infection is rational.

Previous studies used large numbers of probiotic species and strains, and their immunomodulatory effects were strain-specific^100^. For example, Youn et al.^84^ reported that the protective effects against influenza virus infection significantly varied among *Lactobacillus* strains. Therefore, the effective strains of probiotics should be selected on the basis of animal and human studies.

Concerning the required quantity of probiotics, they must be consumed in sufficient quantities (>7 log CFU) to have protective and curative effects against respiratory tract infections including COVID-19. Microencapsulation should be used to protect probiotics against harsh conditions to permit their accumulation at attachment sites in the intestine at sufficient levels. Other factors associated with the beneficial effects of probiotics in preventing or treating diseases such as COVID-19 including the time of administration in the context of disease, the probiotics dose, treatment duration, and the health and microbiota status of the host. Clinical trials considering these factors should be launched in the near future.

It has been demonstrated that the numbers and biodiversity of GI microbes usually decreases with age and antibiotics therapy. This dysbiosis has been remarkably linked to several infectious, metabolic, or inflammatory diseases and conditions such as malnutrition, colon cancer, obesity, diabetes, and atherosclerosis^101^. Patients with disordered microbiota and the elderly are the most susceptible to COVID-19. Therefore, probiotics supplementation in those groups could likely improve the ability of the GI microbiota to modulate immune activity and thus prevent viral infections including COVID-19.

In a meta-analysis of 52 published studies that investigated the ability of probiotics to prevent or treat several disorder conditions, the strongest evidence concerning the efficiency of probiotics was observed for five diseases including acute respiratory tract infections^102^. In another meta-analysis, King et al.^103^ evaluated 17 randomized controlled trials that assessed the preventive effects of probiotics against acute lower digestive tract infections, acute respiratory tract infections, or acute otitis media in infants and/or children. Probiotics comprising single or combinations of *Lactobacillus* and *Bifidobacterium* strains were delivered to the target sample via food or supplements for 4 days to 9 months. Children who were treated with probiotics had a lower risk of requiring antibiotic prescriptions than untreated children. Therefore, probiotics may reduce the risk of common acute infections and thus reduce antibiotic use in infants and children. Further, the effects of probiotics on respiratory tract infections were investigated in a meta-analysis of 23 randomized controlled trials involving 6269 children. The results illustrated that probiotic consumption significantly reduced the severity of symptoms of infected children and the duration of infection^104^.

Research is needed to determine the accurate mechanisms of action of probiotics against coronaviruses including SARS-CoV-2 in healthy or infected animal models. These studies may lead to a better understanding of the bacterial dynamics in the GI tract.

Animal or human studies could be used to assess the direct effects of intranasal probiotics through targeting pathogens in the lungs and indirect effect occurring through the modulation of immune activity. These studies may be helpful for treating viral infections such as COVID-19.

To date, the health effects of probiotics have been attributed to various activities including their ability to support intestinal integrity and maintain intestinal permeability, competition with pathogens for nutrients and attachment sites, the regulation of immune cell activity against invading pathogens, and the prevention of excessive immune responses and inflammation^100^. Further studies are needed to examine the activity of probiotics against different coronaviruses such SARS-CoV-1, SARS-CoV-2, and MERS-CoV to fully understand their underlying mechanisms against viruses. Studies are also required to determine any adverse effects of probiotic supplementation.

## Conclusions

Probiotics are live microorganisms that confer health benefits when consumed in adequate amounts, including enhanced immune activity and the clearance of respiratory tract infections. It is evident that probiotics can reduce the incidence and severity of diseases, suggesting their promise for treating or preventing COVID-19. Probiotics could help prevent COVID-19 by maintaining the human GI or lung microbiota because dysbiosis plays a major role in the susceptibility of people to infectious diseases. In vitro and clinical studies are required to examine the potential preventive and curative effects of probiotics against SARS-CoV-2 infection.

## Data Availability

The authors confirm that all data are available within the article.
